# Placenta Percreta in First Trimester after Multiple Rounds of Failed Medical Management for a Missed Abortion

**DOI:** 10.1155/2017/6070732

**Published:** 2017-10-18

**Authors:** Jaimin Shah, Eduardo Matta, Fernando Acosta, Natalia Golardi, Cristina Wallace-Huff

**Affiliations:** ^1^Department of Obstetrics, Gynecology and Reproductive Sciences, McGovern Medical School, The University of Texas Health Science Center at Houston, Houston, TX, USA; ^2^Department of Diagnostic and Interventional Imaging, McGovern Medical School, The University of Texas Health Science Center at Houston, Houston, TX, USA; ^3^Department of Pathology and Laboratory Medicine, McGovern Medical School, The University of Texas Health Science Center at Houston, Houston, TX, USA

## Abstract

**Background:**

The detection of a morbidly adherent placenta (MAP) in the first trimester is rare. Risk factors such as multiparity, advanced maternal age, prior cesarean delivery, prior myomectomy, placenta previa, or previous uterine evacuation place patients at a higher risk for having abnormal placental implantation. If these patients have a first trimester missed abortion and fail medical management, it is important that providers have a heightened suspicion for a MAP.

**Case:**

A 24-year-old G4P3003 with 3 prior cesarean deliveries underwent multiple rounds of failed medical management for a missed abortion. She had a dilation and curettage that was complicated by a significant hemorrhage and ultimately required an urgent hysterectomy.

**Conclusion:**

When patients fail medical management for a missed abortion, providers need to assess the patient's risk factors for a MAP. If risk factors are present, a series of specific evaluations should be triggered to rule out a MAP and help further guide management. Early diagnosis of a MAP allows providers to coordinate a multidisciplinary treatment approach and thoroughly counsel patients. Ensuring adequate resources and personnel at a tertiary hospital is essential to provide the highest quality of care and improve outcomes.

## 1. Introduction

The detection of a morbidly adherent placenta (MAP) in early pregnancy is rare. Routine first trimester transvaginal ultrasounds (TVUS) usually do not focus on localization and implantation of the placenta [1]. Generally, a MAP is not clinically detected until later in pregnancy [2]. Risk factors such as multiparity, advanced maternal age, prior cesarean delivery, prior myomectomy, placenta previa, or previous uterine evacuation place patients at a higher risk for having abnormal placental implantation in future pregnancies [2, 3]. The incidence of MAPs in early gestation has been increasing likely due to the rising rates of cesarean deliveries and prior uterine surgery [1, 4–7]. In patients with risk factors for abnormal placental implantation and who fail medical management for a first trimester abortion, it is important that providers have an increased suspicion for a MAP. We report a case of a patient with a 7-week missed abortion with three prior cesarean deliveries that failed multiple rounds of medical management. She subsequently had an attempted dilation and curettage that was complicated by a significant hemorrhage and she ultimately required an urgent hysterectomy.

## 2. Case

A 25-year-old G4P3003 at 7 weeks and 1 day by last menstrual period with a medical history of 3 previously documented low transverse cesarean deliveries and obesity (BMI: 34) presented for management at a county hospital for a missed abortion diagnosed at an outlying rural clinic. The patient reported that this was a planned pregnancy and desired future pregnancies; she denied any spotting, cramping, or passage of any tissue. The formal TVUS report at the outside clinic showed an intrauterine pregnancy with a 27.8 mm mean sac diameter consistent with 8 weeks and no fetal cardiac activity seen; no evidence of a MAP was noted in the report. A repeat bedside TVUS in clinic by a resident physician showed an irregular shaped gestational sac with a crown rump length of 1.5 cm.

After thoroughly counseling the patient on expectant, medical, and surgical management, she elected for medical management. The patient was uninsured and declined surgical management as she did not want to incur the expense of the procedure. The patient received 800 mcg of misoprostol per vagina (PV) in clinic and she was sent home with a prescription to take two additional doses of 800 mcg buccally every 24–48 hours if needed until she noted passage of clots or tissue. She was instructed to return to clinic in one week unless she developed heavy bleeding soaking greater than two pads an hour for two hours or fever greater than 100.4 degrees Fahrenheit [8]. She followed up one week later and denied spotting, cramping, or passage of tissue. A bedside TVUS by a resident physician showed no change from the week prior. Given that the patient's insurance eligibility status was still pending, the patient declined surgical management due to the potential financial burden and declined expectant management. The patient was counseled that no data supports multiple rounds of medical management but, given her insurance eligibility status and strong desire to not incur surgical fees, she received two more rounds of medical management without resolution of her missed abortion. The patient was then able to acquire insurance approval. She then opted for surgical management. She presented three days later to the ambulatory outpatient surgical center.

She was Rh positive with a hemoglobin of 12.7 g/dL. During her procedure, the cervix was dilated followed by insertion of the suction curette; some products of conception were evacuated but the canister filled quickly with bright red blood. Upon removing the curette, she continued to bleed heavily. Methergine was administered intramuscularly which helped decrease the amount of bleeding. The estimated blood loss (EBL) was 1200 cc; two units of packed red blood cells (PRBC) were given and the main operating room (OR) and hospital were notified for her immediate transfer since the ambulatory surgery center was not sufficiently equipped for this level of care. A foley balloon was placed into the uterus and inflated to 60 cc. This was able to tamponade and minimize the bleeding.

The patient was transferred to the main hospital by ambulance. A TVUS was performed which showed products of conception versus a 5 cm hematometra. Given that the patient had refractory abdominal pain unrelieved by intravenous morphine and a concern for an expanding hematometra, the patient was taken back to the OR for an exploratory laparotomy. The patient was consented for a possible total abdominal hysterectomy versus evacuation of hematometra. Upon entry into the abdomen, dense abdominal adhesions were noted; there was approximately 200 cc of hemoperitoneum in the rectouterine pouch. It was noted that there was a 7-8 cm portion of the lower uterine segment that displayed placental tissue overlying the uterine serosa by 1 mm. The decision was made to proceed with a hysterectomy. She received 1 unit each of fresh frozen plasma and PRBC intraoperatively. The EBL intraoperatively was 500 cc bringing the total blood loss to 1900 cc. A cystoscopy was performed and bladder involvement was ruled out. The patient met all postoperative milestones and recovered well.

**Figure 1:**
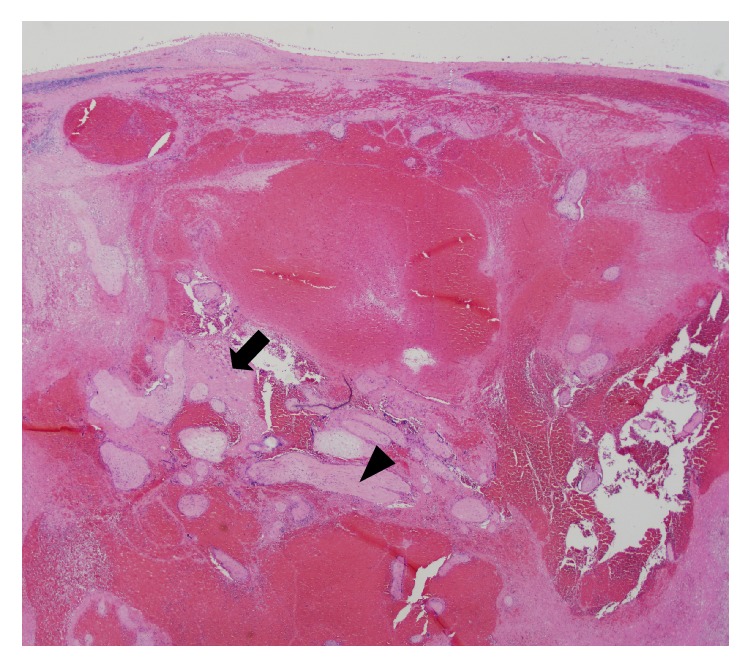
Necrotic myometrium (arrow) with degenerating chorionic villi (arrowhead) transecting through the entire myometrial thickness to the serosal surface with extensive hemorrhage (H&E stain, 20x magnification).

Final pathology showed a placenta percreta. Sectioning through the patient's myometrium showed extensive hemorrhage dissecting through the entire myometrial thickness at the level of the lower uterine segment (Figure 1). Microscopic evaluation showed numerous chorionic villi penetrating through the entire thickness of the myometrial wall and through the uterine serosa which is diagnostic of a placenta percreta [9].

## 3. Discussion

Upon review of the literature, a MAP is a rare finding to detect in the first trimester. Of the MAPs, placenta accreta occurs 75%, placenta increta 18%, and placenta percreta 7% of the time [2, 5]. To our knowledge, there have been 26 prior MAPs diagnosed in the first trimester and treated before 15 weeks' gestation (Table 1). Most patients had a history of a prior cesarean delivery leaving possible scar tissue in the anterior uterine wall [1, 4]. Twenty-two patients required a hysterectomy while four had conservative management and retained their uterus. The majority of patients had a risk factor for a MAP but a few cases occurred in patients with a nonscarred uterus [1]. It is our understanding there were only two other cases that attempted one round of medical management in which both cases ended up with an hysterectomy [3, 10]. Per The American College of Obstetricians and Gynecologists (ACOG), they recommend one round of medical management for missed abortions and then consider alternate management options [8]. Our patient did not have insurance coverage so the option of surgery after her failed first round of medical management was not financially feasible. Given patient's socioeconomic constraints, offering continued trials of medical management could still be within reason with strict precautions. Providers need to consider clinical nuances, patient treatment preferences, and compliance with treatment regimen.

However, failed medical management raises concern, especially in patients with risk factors for a MAP. This should trigger further evaluation with a thorough repeat formal TVUS to rule out a MAP and have radiology look closer at the placenta to myometrial interaction and morphology. Relaying identifiable risk factors and pertinent clinical findings to radiology is important to assist in their assessment. Ultrasound is the primary modality for diagnosing a MAP [11]; a MAP is more difficult to diagnosis in the first trimester with much lower accuracies compared to second and third trimesters [1, 12]. On ultrasound, some features indicative of a MAP include thinning or nonvisualization of the myometrium overlying the placenta, presence of placental lacunae (irregular shaped vascular spaces) with turbulent flow, loss of retroplacental clear space, interruption of the interface between the bladder and myometrium, and hypervascularization of the placental-myometrial interface [5, 11–14]. Measurement of the smallest anterior myometrial thickness in a sagittal view combined with the number of prior cesarean deliveries has been shown to significantly increase the prediction of a MAP [13]. In addition, patients with risk factors for a MAP should have imaging of the anterior myometrium and bladder with a high-frequency transducer. A similar approach should be taken if a placenta previa or loss of the retroplacental clear space is detected.

**Figure 2:**
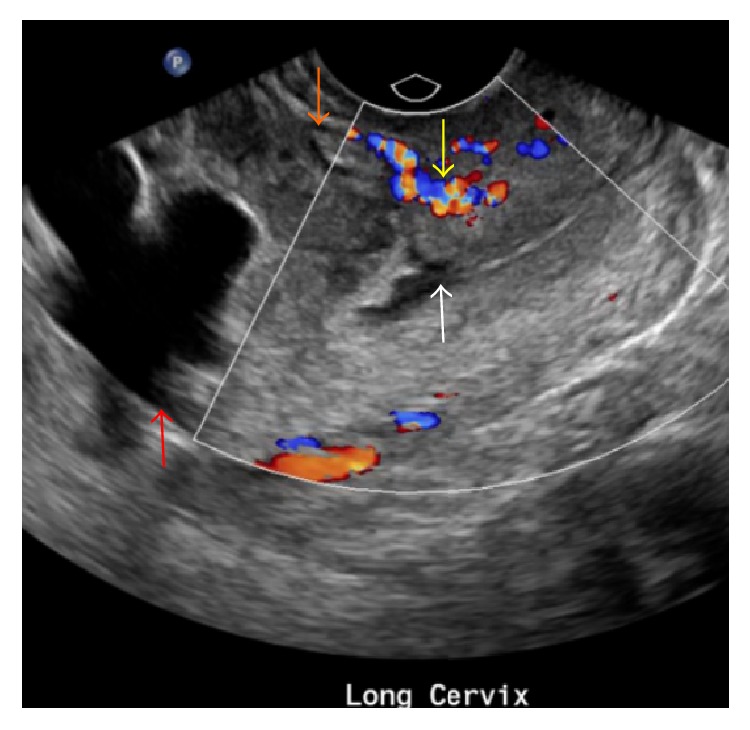
Longitudinal grayscale and color Doppler image of the lower uterine segment and cervix demonstrates endometrial fluid (red arrow) and an irregular, heterogeneously hypoechoic placenta (orange arrow) with blood supply from cervical vessels (yellow arrow) extending into the anterior myometrial wall and over the internal cervical os (white arrow).

Vascular findings have also been described in a MAP. Placental lacunae and indistinct intraplacental channels with turbulent flow have the highest sensitivity for a MAP [11, 15]. These should not be confused with vascular lakes, which are more round and have laminar flow. While retroplacental hypervascularity can occur with a MAP, disruption of flow may be seen at the site of invasion [16]. Moreover, multiple enlarged vessels can surround the myometrium in cases of placenta percreta, which may also be associated with an irregular vascular bladder wall [14]. Upon review of our patient's TVUS images with our radiology team after they were aware of the diagnosis, they retrospectively noted potential features that were indicative of a MAP (Figure 2).

Magnetic resonance imaging (MRI) may be used when an ultrasound is not definitive or if the placenta is posterior [16]. MRI protocols include a form of T2-weighted imaging, where the placenta is distinct from the myometrium and homogeneous, except for a thin septae. MRI findings of a MAP include uterine bulging, heterogeneous placenta, thick T2-dark intraplacental bands, and focal disruption of the myometrium. However, myometrial thinning can be misleading and may be normal. In cases of placenta percreta, direct invasion or tenting of the bladder may be present [14].

In patients with a concern for a first trimester MAP, their management needs to entail extensive counseling regarding therapeutic options with a definitive (hysterectomy) or conservative (leave placenta in situ) management depending on patients fertility goals [5, 11]. Preoperative counseling for these patients ought to include the potential for a hysterectomy, risk of a hemorrhage requiring blood transfusions, and maternal death in order for patients to have realistic expectations about the various outcomes [5, 11]. If there is a high suspicion for a first trimester MAP, presurgical planning along with a multidisciplinary approach is essential to help prevent complications at the time of surgery; appropriate specialties need to be consulted and the operating room should be appropriately prepped (e.g., blood products and necessary instruments) [3, 5]. A prior study has recommended focusing on patient education to help increase the detection of first trimester MAPs; at the time of discharge after a cesarean delivery, it is important to discuss with patients that in future pregnancies an early prenatal visit with TVUS to rule out a MAP should be performed [4].

Being adequately prepared in the OR with appropriate hospital resources is essential. Our patient was scheduled for a routine minor procedure in an outpatient ambulatory surgical center but necessitated transfer to the main hospital for higher level of care. In patients with risk factors and possible concern for a MAP, it is critical that these surgeries be performed in a tertiary hospital setting where sufficient resources are available if any complications arise [11].

MAPs represent a life-threatening concern and pose additional risk when patients are not diagnosed until the time of surgery. In patients with failed medical management for a missed abortion, assessment of MAP risk factors is critical and considered to further guide management. Communicating pertinent information to radiology better equips them in their ultrasound investigation and may place additional focus on detecting MAPs earlier and prevent unanticipated discoveries peripartum [12]. Early diagnosis of a MAP allows providers to coordinate a multidisciplinary treatment approach and thoroughly counsel patients on options and expectations. Ensuring adequate resources and personnel at a tertiary hospital is necessary to provide the highest quality of care and improve outcomes.

## Figures and Tables

**Table 1:** Morbidly adherent placentas (MAPs) diagnosed in the first trimester and treated before 15 weeks' gestation [1, 3, 4].

Author and year	Type of MAP	Prior CS^§^	Prior D&C^†^	GA at Diagnosis^#^	Presenting symptoms	US diagnostic of MAP	Management & outcome
Helkjaer et al., 1982	n/a^∧^	1	0	11 wks	VB^*∗*^	n/a	Laparotomy, repair
Woolcott et al., 1987	Percreta	2	0	10 wks	VB	No; missed abortion	Laparotomy, hysterectomy + bladder repair
Haider, 1990	Percreta	1	0	10 wks	VB	No; missed abortion	Laparotomy, hysterectomy
Ecker et al., 1992	Increta	n/a	n/a	1st trimester	n/a	n/a	Laparotomy, hysterectomy
Arredondo et al., 1995	Accreta	0	3	1st trimester	None	No; missed abortion	Laparotomy, hysterectomy
Gherman et al., 1999	Increta	1	1	8 wks	VB/abd pain	Yes; suspected MAP	Laparoscopy/laparotomy, hysterectomy
Walter et al., 1999	Increta	1	0	11 wks	VB	n/a	D&C/laparotomy, hysterectomy
Marcus et al., 1999	Percreta	2	0	13 wks	VB	n/a	UAE^*∗∗*^/D&C/laparotomy, hysterectomy
Chanrachakul et al., 2001	Increta	1	0	7 wks	VB	No; missed abortion	D&C/laparotomy, hysterectomy
Hopker et al., 2002	Percreta	1	1	10 wks	Abd pain	Yes; suspected MAP versus invasive mole	Laparotomy, hysterectomy
Shih et al., 2002	Accreta	0	0	8 wks	VB	Yes; suspected MAP	Elective laparotomy at 15 wks, hysterectomy
Buetow, 2002	Percreta	1	0	1st trimester	VB/pelvic pain	Yes; suspected MAP	Laparotomy, hysterectomy
Chen et al., 2002	Accreta	2	1	9 wks	VB	Yes; suspected MAP	Laparotomy, hysterectomy
Liang et al., 2003	Percreta	2	n/a	1st trimester	Abd pain/shock	n/a	Laparotomy, hysterectomy
Liu et al., 2003	Increta	1	0	1st trimester	n/a	n/a	UAE/laparotomy, hysterectomy
Coniglio and Dickinson, 2004	Accreta	2	0	8 wks	Abd pain/shock	Yes; suspected Cesarean scar pregnancy	Laparotomy, repair
Dabulis and McGuirk, 2007	Percreta	3	1	9 wks	Abd pain	Yes; suspected MAP	Laparotomy, hysterectomy
Son et al., 2007	Increta	0	3	8 wks	Abd pain/syncope	Abd/pelvis computed tomography	Laparotomy, hysterectomy
Tanyi et al., 2008	Percreta	1	1	7 wks	VB/abd pain	No; threatened abortion	D&C/laparotomy, hysterectomy
Papadaskis et al., 2008^††^	Percreta	2	1	11 wks	VB	No; missed abortion	D&C/laparotomy, hysterectomy
Soleymani et al., 2009	Increta	0	0	11 wks	VB	Yes; suspected MAP	D&C/UAE, resolved
Yang et al., 2009	Increta	2	3	12 wks	VB	Yes; suspected MAP	UAE, resolved
Pont et al., 2010	Percreta	1	1	13 wks	Abd pain	n/a	Laparotomy, hysterectomy
Hanif et al., 2011	Percreta	2	2	12 wks	Abd pain/syncope	No; ectopic pregnancy	Laparotomy, hysterectomy
Shojai et al., 2012^††^	Increta	2	0	7 wks	n/a	No; missed abortion	D&C/laparotomy, hysterectomy
Shaamash et al., 2014	Accreta	2	0	11 wks	VB/abd pain	Yes; suspected MAP versus molar changes	D&C/laparotomy, hysterectomy

^§^Cesarean delivery; ^†^dilation and curettage; ^*∗*^vaginal bleeding; ^*∗∗*^uterine artery embolization; ^#^gestational age;  ^∧^unknown or not reported; ^††^failed medical management with misoprostol.
